# Ultrasonic sculpting of virtual optical waveguides in tissue

**DOI:** 10.1038/s41467-018-07856-w

**Published:** 2019-01-09

**Authors:** Maysamreza Chamanzar, Matteo Giuseppe Scopelliti, Julien Bloch, Ninh Do, Minyoung Huh, Dongjin Seo, Jillian Iafrati, Vikaas S. Sohal, Mohammad-Reza Alam, Michel M. Maharbiz

**Affiliations:** 1 Electrical and Computer Engineering Department, Carnegie Mellon University, Pittsburgh, 15213 PA USA; 2 Electrical Engineering and Computer Science Department, University of California, Berkeley, 94720 CA USA; 3 Mechanical Engineering Department, University of California, 94720, Berkeley, CA USA; 4 Department of Psychiatry, University of California, San Francisco, 94103 CA USA; 5 Center for Integrative Neuroscience, University of California, San Francisco, 94158 CA USA; 6 Bioengineering Department, University of California, Berkeley, 94720 CA USA; 7 Center for Neural Engineering and Prostheses, University of California, Berkeley, 94720 CA USA; 8 Chan Zuckerberg Biohub, San Francisco, 94158 CA USA

## Abstract

Optical imaging and stimulation are widely used to study biological events. However, scattering processes limit the depth to which externally focused light can penetrate tissue. Optical fibers and waveguides are commonly inserted into tissue when delivering light deeper than a few millimeters. This approach, however, introduces complications arising from tissue damage. In addition, it makes it difficult to steer light. Here, we demonstrate that ultrasound can be used to define and steer the trajectory of light within scattering media by exploiting local pressure differences created by acoustic waves that result in refractive index contrasts. We show that virtual light pipes can be created deep into the tissue (>18 scattering mean free paths). We demonstrate the application of this technology in confining light through mouse brain tissue. This technology is likely extendable to form arbitrary light patterns within tissue, extending both the reach and the flexibility of light-based methods.

## Abstract (web summary)

Optical imaging of tissue is a powerful technique but delivering light deep into scattering tissue is not possible without using invasive methods. Here, the authors demonstrate that patterned ultrasound can define and control the trajectory of light in tissue using pressure-induced index contrasts.

## Introduction

The advent of optical reporters for the optical stimulation and in vivo imaging of biological events was a major breakthrough, especially as applied in the central nervous system for high throughput functional recording and stimulation of brain activity with high temporal resolution^1–7^. All existing techniques, however, suffer from an inability to deliver light deep into tissue with high spatial resolution^6,8^. Additionally, in the context of the central nervous system, which harbors widely distributed neural processes, system-wide interrogation requires either fast optical beam-steering capability or simultaneous multi-site illumination. As light propagates through tissue, it undergoes diffraction, scattering, and absorption; as a result, the beam widens and the intensity of light rapidly falls below the threshold of excitation of opsins and optical tags. This limits optical techniques to superficial layers of the tissue (100’s of micrometers)^9^. To alleviate these issues, either the intensity of light on the surface of the brain must be increased, which can be detrimental to the tissue, or invasive light guides such as optical fibers or waveguides need to be inserted into the tissue^6,7,10,11^. Steering light to different locations within the tissue using implantable waveguides is difficult and invasive, limiting common optical imaging and stimulation techniques to fixed positions in the tissue. Wavefront correction techniques have also been used to counterbalance the random scattering in opaque tissue and deliver coherent light deeper into the medium^12,13^. Such methods require a priori feedback from the scattered pattern of light within the tissue.

**Fig. 1:**
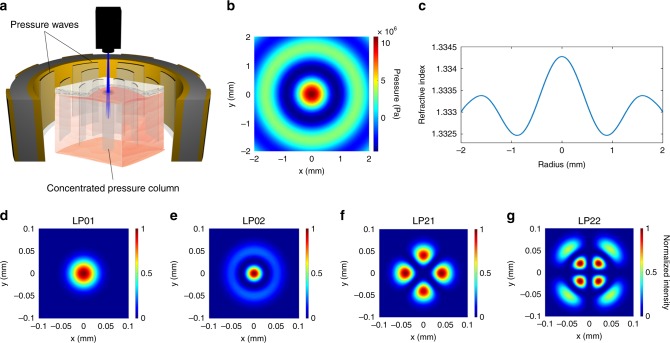
Ultrasound sculpts virtual optical waveguides. **a** Schematic illustration of acousto-optic waveguide formation in a tissue. Pressure-induced density gradients in the tissue result in a refractive index contrast profile that forms optical waveguides through which light can be confined. **b** Standing wave ultrasonic resonant mode of an infinite cylindrical transducer (radius = 19 mm, thickness = 3 mm) in water medium (FEM simulation result). The pressure intensity is shown with the Pascal units. **c** Refractive index profile calculated at a radial cross-section of the cylindrical waveguide. **d**–**g** The intensity profiles of a subset of confined optical modes calculated using finite difference mode analysis. In each case, the intensity is normalized to its maximum

Here, we demonstrate that non-invasive ultrasonic waves can be used to confine and steer light deep (a few millimeters) into tissue without having to insert a physical light guide. The concept of acousto-optic confinement is shown schematically in Fig. 1a. Acoustic waves propagating in a tissue compress and rarefy the tissue and change its density locally. The medium is compressed at the intensity peak of the acoustic wave and this results in an increase in the local refractive index. Conversely, the medium is rarefied near the troughs and the refractive index is decreased^14^. Optical waves can be confined within high refractive index regions if these are flanked by adjacent low index regions, thereby forming an optical waveguide (Fig. 1a); this is the basic principle behind optical waveguides such as optical fibers. Similar acousto-optic phenomena have been traditionally employed in acousto-optic tomography^15,16^, optical modulators via acousto-optic crystals^17,18^, tunable optical filters^19,20^, tunable lenses^21,22^, and optical deflectors^23,24^. In this work, we show that the target medium (e.g., the tissue) itself can be modulated by ultrasonic standing waves to confine and guide light.

## Results

### Ultrasonic standing waves sculpt optical modes in the medium

Piezoelectric transducers convert electrical energy to mechanical vibrations that generate acoustic waves in a medium. Such acoustic waves are widely used for clinical imaging at ultrasonic frequencies and can propagate into the tissue with minimal scattering and absorption (propagation loss in tissue is ~0.3–0.6 dB cm^−1^ MHz^−1^)^25^. We employ an ultrasound transducer array to produce standing waves within a cylindrical cavity at discrete resonance frequencies, for which the location of peaks and troughs are fixed in space. The cavity walls are made from piezoelectric ceramics that vibrate to generate ultrasound resonance modes, which possess well-defined spatial patterns. The pressure amplitude profile of a cylindrical ultrasonic cavity at the resonance frequency of 1.0235 MHz is shown in Fig. 1b for a 13 V drive voltage; note the pressure maximum at the center (see Methods). As expected, the refractive index profile is a function of the ultrasonic standing wave pressure (the radial cross-section is shown in Fig. 1c); a maximum refractive index contrast of 1.8 × 10^−3^ is achieved. Given this refractive index contrast, the standing waves support multiple confined optical guided modes, some of which are shown in Fig. 1d–g, calculated using a finite difference mode analysis method (http://www.lumerical.com/tcad-products/mode/). This ultrasonically sculpted optical waveguide is multimode and the modes are similar to those of a graded index (GRIN) fiber^26–28^. GRIN fibers are made by doping the fiber core material to change the refractive index along the radial direction with a maximum in the center. The most common radial refractive index profile in a GRIN fiber is a parabolic profile, with a typical maximum refractive index contrast of Δ*n* = 2 × 10^−2^ that results in a numerical aperture of NA = 0.24 (refs. ^26–28^). However, a major difference between a traditional GRIN fiber and our ultrasonically sculpted waveguides is that the GRIN fibers are made of minimally scattering materials, whereas the ultrasonically sculpted waveguides are realized in tissue, which is a scattering medium. Therefore, the ultrasonically-defined optical waveguides within the tissue support leaky modes. As a consequence, the confined and guided modes are partially coupled to radiation modes, contributing to a larger propagation loss compared to a traditional GRIN fiber. Nevertheless, our experimental results demonstrate confinement and waveguiding through the sculpted waveguides.

Another difference between the in-tissue ultrasonically sculpted optical waveguides and traditional GRIN fibers is the smaller numerical aperture (NA = 0.0694 due to the smaller refractive contrast Δ*n* = 0.001808. Despite this smaller refractive index contrast, the formed optical waveguide supports confined guided modes. The fundamental guided mode (Fig. 1d) is very well confined in the central high-pressure region of the ultrasonic wave and has an effective index of *n*_eff_ = 1.3342. The full width at half maximum (FWHM) of the fundamental mode is 67.6 μm, which is much smaller than the FWHM  of the central lobe of the refractive index profile (i.e., 876.7 μm).

The high-pressure regions of the ultrasonic standing wave shown in Fig. 1b oscillate in time; every half-cycle, the peak positive pressure becomes peak negative pressure. When attempting to exploit the pressure peaks in the cavity, the input light needs to be pulsed and synchronized with the positive peak pressure of the ultrasonic wave. This keeps light guided in the central ultrasonically sculpted optical waveguide. The temporal dynamics of light (~2 femtosecond) are much faster than that of ultrasound (~1 μs); by pulsing the input light at the same frequency of ultrasound waves but with a duty cycle of 10%, photons are guided through the central waveguide for ~100 ns every 1 μs.

**Fig. 2:**
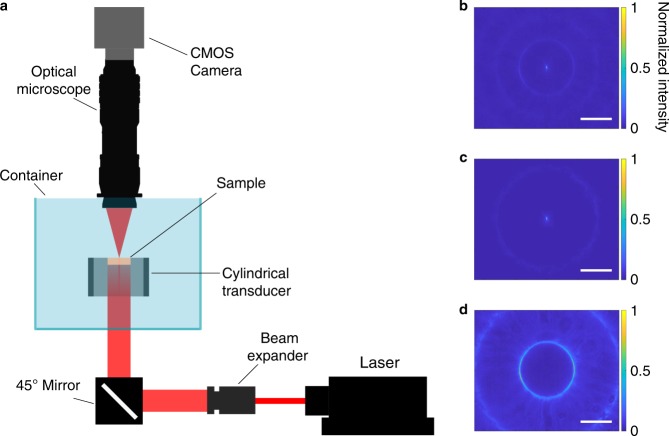
Light is confined by pressure patterns. **a** Schematic of the characterization setup. Experimental 2D cross-section image of the optical beam profile **b** with ultrasound and laser light not synchronized, **c** with ultrasound and laser light synchronized and phase locked at +90° to form only the central waveguide and **d** with the phase shift at −90° to form only the ring waveguide. The intensity of each 2D cross-section is normalized to its maximum value. The scale bar is 1 mm

To quantify the extent and depth of optical confinement in the cylindrical cavity (19 mm radius, 30 mm height), an expanded and modulated laser beam at λ = 650 nm was collimated into a DI water tank containing the cylindrical ultrasonic cavity (Fig. 2a). The transducers were driven by pulsed electrical signals generated by a commercial waveform generator (Keysight 33522B, Keysight Technologies, USA) and amplified by a linear RF power amplifier (ENI A300, Electronics & Innovation Ltd., USA). A custom designed water-immersion microscope was used to image the 2D cross-section of the optical beam profile from the top in the transmission path (see Methods and Supplementary Fig. 1).

Figure 2b shows a transverse optical beam profile at the resonance frequency of 1.028 MHz when the laser and ultrasound are not synchronized. Both the center waveguide and the first and the second ring waveguides corresponding to the high-pressure rings surrounding the central pressure peak are shown. The peaks and troughs of the standing pressure waves oscillate with time. The first ring in the pressure profile is completely out of phase with respect to the central pressure peak (Fig. 1b). When the center is at the highest positive pressure, the first ring is at the highest negative pressure and cannot confine light. To verify this, we pulsed the laser in synchrony with the ultrasound. When the laser modulation was phase locked with the ultrasound at a +90° phase shift, only the central waveguide guided the light beam (Fig. 2c). Conversely, when the laser modulation was phase locked at −90°, only the first ring waveguide was present (Fig. 2d).

### Confining light in scattering media using ultrasound

To characterize ultrasonically sculpted optical waveguides in scattering media, we first characterized homogeneous scattering tissue phantoms made of different concentrations of Intralipid 20% emulsion in an agar gel host matrix (see Methods)^29^. The level of scattering in tissue phantom samples discussed in this paper can be quantitatively characterized using the scattering coefficient, *μ*_s_ and the optical thickness (*OT*). It is common to define a reduced scattering coefficient as $\mu _{\mathrm{s}}^\prime = \mu _{\mathrm{s}} \times \left( {1 - g} \right)$, where *μ*_s_ is the scattering coefficient and *g* is the anisotropy factor that characterizes the directionality of the scattered photons. In most biological samples, *g* = 0.9 (ref. ^30^). The scattering mean free path, defined as $\ell {\mathrm{s}} = 1/\mu _{\mathrm{s}}$ is the average path length between two successive scattering events. The transport mean free path (TMFP), defined as $\ell ^ \ast = 1/\mu _{\mathrm{s}}^\prime$ is the average distance beyond which the direction of photons can be assumed to be effectively random. The optical thickness, *OT*, is defined as the geometrical thickness of the tissue phantom divided by the scattering mean free path, i.e., $\ell {\mathrm{s}}$.

**Fig. 3:**
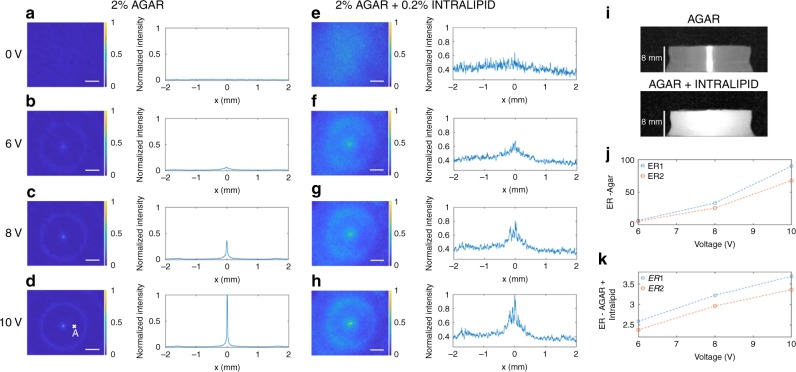
Light is confined within tissue phantoms. Optical beam profile imaged experimentally 100 μm below the top surface of the tissue phantom in agar and in a scattering tissue phantom. **a**–**d** 2D cross-sections of the optical beam profile and radial cross-section plots in 2% agar when ultrasound is: **a** off, **b** driven at 6 V, **c** 8 V, and **d** 10 V. In each case, the intensity has been normalized to the maximum intensity of (**d**). **e**–**h** 2D cross-sections of the optical beam profile and radial cross-section plots in a scattering tissue phantom composed of 2% agar mixed with 0.2% Intralipid when ultrasound is: **e** off, **f** driven at 6 V, **g** 8 V, and **h** 10 V. In each case, the intensity has been normalized to the maximum intensity of (**h**). Side view of agar and the scattering tissue phantoms shown in **i** when illuminated by a narrow (2 mm) collimated beam of laser. Extinction ratios measured according to the definition of Extinction Ratio 1 (ER1) (blue) and Extinction Ratio 2 (ER2*)* (orange) in: **j** 2% agar and in **k** the scattering tissue phantom. The scale bar is 1 mm

The ultrasonically sculpted optical waveguide was first imaged in a homogeneous agar sample (2% agar) in the form of a cylindrical phantom (13.55 mm radius and 8 mm height). 2D cross-sectional images of the optical beam profile were captured in transmission mode for different input drive voltages (Fig. 3a–d): laser beam is coupled in from the bottom (*z* = −8 mm) and the output is imaged on the top side of the tissue phantom (slightly below the top surface at *z* = -0.1 mm), just 100 µm below the surface to avoid inconsistencies of the facet. As the drive voltage increases and the pressure wave intensity increases, the waveguide becomes narrower. By increasing the pressure, the refractive index contrast becomes larger, which results in increased confinement of the waveguide modes. To demonstrate the application of this method for confining and guiding light within a scattering medium, we prepared a rod-shape tissue phantom (13.55 mm radius and 8 mm height) composed of 2% agar mixed with 0.2% Intralipid as the scattering agent. This tissue phantom has a reduced scattering coefficient of $\mu _{\mathrm{s}}^\prime = 2.35\,{\mathrm{cm}}^{ - 1}$, measured using the oblique incidence reflectometry (OIR) technique^31^. This 8 mm tissue phantom has an optical thickness of 18.8 scattering mean free paths (i.e., *OT* = 18.8 ℓs). Since the optical thickness of the medium at the wavelength of illumination is much larger than the scattering mean free path length (i.e., ℓs), light is confined in a regime where at least a few successive scattering events happen. 2D cross-sectional images of the optical beam profile are shown in Fig. 3e–h at different input drive voltages. When a narrow beam of laser impinges upon this tissue phantom, the whole phantom is illuminated due to the high level of scattering (Fig. 3i).

To quantify the enhancement achieved using ultrasound to form an optical waveguide to guide ballistic photons within the scattering tissue, we define two figures of merit, Extinction Ratio 1 (ER1) and Extinction Ratio 2 (ER2). ER1 is the extinction ratio defined as the maximum intensity of light in the waveguide core divided by the intensity of light at the location of the first ring (point A in Fig. 3d). As discussed earlier, when the pressure in the central spot is at the positive peak value, the pressure at the first ring is at the negative peak value. As a result, the medium is compressed in the center region and rarefied in the surrounding region. The refractive index of the medium is slightly increased in the center region and slightly decreased in the surrounding region, thus photons are confined in the center region due to the induced refractive index contrast. This pronounced contrast is quantified by ER1. On the other hand, ER2 is the extinction ratio defined as the maximum intensity of the waveguide mode divided by the background intensity at the same location when ultrasound is off. These two figures of merit are compared for different input voltages in agar sample (Fig. 3j) and the scattering tissue phantom sample (Fig. 3k). As expected, optical confinement is lessened with more scattering. Taken together, these results demonstrate that ultrasonically sculpted optical waveguides can confine and guide light through an optically thick scattering tissue phantom (~8 mm, OT ~18.8 ℓs) with an extinction ratio of ER1 ≈ 3.7. The extinction ratio increases and the spot size decreases as we increase the input voltage.

**Fig. 4:**
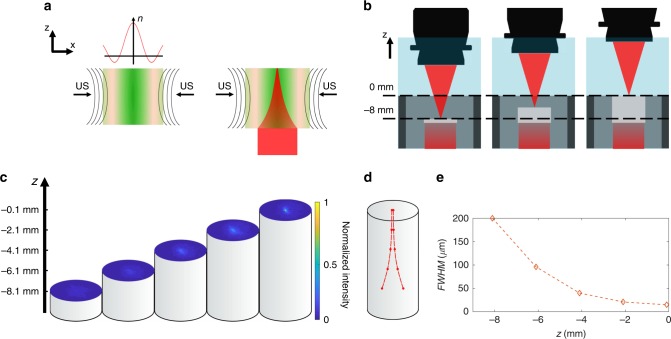
Gradual beam axial confinement. **a** Ultrasonic waves (US) create a pressure standing wave (green overlay) that modulates the local refractive index of the tissue, sculpting an optical waveguide. **b** Schematic representation of the experiment, the dashed black lines indicate the highest and lowest location of the top facets of the tissue phantoms. **c** Experimentally imaged 2D cross-sections of the optical beam profile propagating inside an agar phantom at different heights along the *z*-axis, showing an effective and gradual confinement of light from *z* = −8.1 mm to *z* = −0.1 mm. **d** The gradual confinement of the beam. In each plane, the dots represent the boundaries of the full width at half maximum of the beam, while the dashed lines show the extrapolated axial trajectory. The intensity has been normalized to the intensity at *z* = −0.1 mm case. **e** Full width at half maximum (FWHM) of the confined optical beam along the axial direction

When ultrasonic waves are launched into the tissue, they create a pressure standing wave that modulates its local refractive index. The tissue is compressed in the central high-pressure region, so its density increases and the local refractive index increases. On the other hand, the negative pressure regions, flanking the central region, are rarefied and as a result both the density and the local refractive index will be decreased (Fig. 4a).

To visualize the optical beam profile of the sculpted waveguide within the ultrasonic cavity, we imaged the output facet of a series of agar phantom rods of different thicknesses. The phantoms were made of 2% agar at 5 different thicknesses of 2, 4, 6, 8, and 10 mm. The phantoms were placed inside a cylindrical ultrasonic transducer so that the input facet is located 10 mm into the transducer at *z* = −10 mm (Fig. 4b). Different tissue phantom rods were successively placed inside the transducer and the output facets were imaged from top in the transmission mode (Fig. 4b). We imaged each sample 100 μm below the top surface (output facet) to reconstruct the evolution of the profile (Fig. 4c). The results show that light is gradually confined to a small spot at the output facet at *z* = −0.1 mm (Fig. 4d). The FWHM of the beam at the input facet (i.e., at *z* = −8.1 mm) is 200 µm, which is then gradually reduced by ~13 times at the output facet (i.e., *z* = −0.1 mm), where it measures only 15 µm (Fig. 4e).

### Steering light using reconfigurable ultrasonic waves

In addition to piping light deep into scattering media, this acousto-optic method can be used to steer light to target different locations within the tissue. Since light follows the pattern of high-pressure regions defined by ultrasound, we can steer the trajectory of light by simply moving or changing the pattern of ultrasound. Moreover, the pattern of ultrasound is also a function of time, and as a result, by changing the phase difference between the pulsed input laser and the ultrasound wave, we can change the pattern of confined light, since photons experience different patterns of high-pressure regions in the tissue at different relative phases. To create a reconfigurable pattern of ultrasound, we need an ultrasonic phased array, where we can independently control the phase and amplitude of each element. In principle, this phased array can consist of a number of piezoelectric elements spatially arranged in any desired shape around the tissue; this is already done for state-of-the-art ultrasonic medical beam-forming^32–34^.

**Fig. 5:**
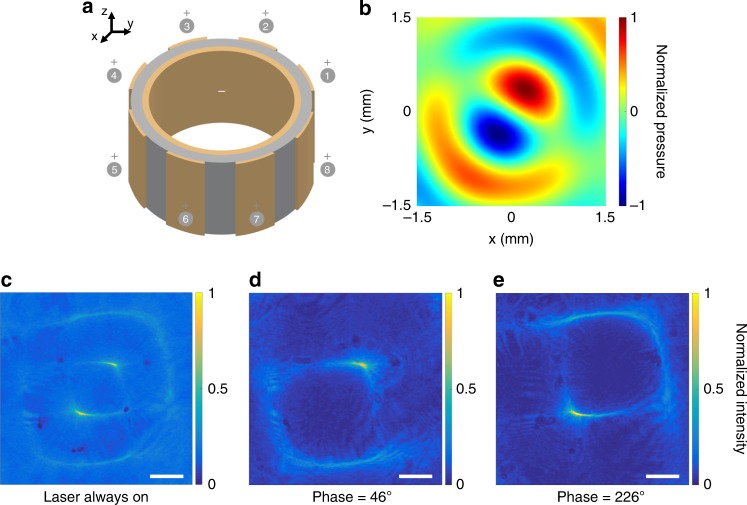
Multi-segment transducer array generates complex light patterns. **a** A multi-segment cylindrical transducer array. Each segment is a cylindrical section with a height of *h* = 30 mm. **b** Simulation of the ultrasonic pressure dipole mode obtained within a cylindrical phased array, where opposite phase voltages are applied to sections 1, 2, 3 and 5, 6, 7. The optical image experimentally captured when: **c** the laser is continuously on (no modulation), **d** the laser is pulsed and synchronized with the ultrasonic transducer at 46° phase, and **e** the laser is synchronized with the ultrasonic transducer at 226° phase. The intensity of each 2D cross-section is normalized to its maximum value and the scale bar is 500 μm

In this paper, we employ an array of 8 piezoelectric transducer elements arranged around a cylindrical cavity (Fig. 5a, *r* = 19 mm). Each element can be independently addressed electrically. The interference between the ultrasound waves launched from different elements results in a standing wave in the region encompassed by the transducer elements; this can be reconfigured by changing the amplitude and phase of the electrical drive signal to each actuator. To demonstrate the optical mode reconfiguration, we discuss a few examples here. Consider an actuation pattern that produces a dipole mode consisting of two out of phase-split lobes (Fig. 5b). Although these split lobes are spatially symmetric, they are interleaved in time (i.e., they happen at different times that correspond to a π phase difference). If the laser is not pulsed and the image is averaged over many cycles of the ultrasound wave, an interference pattern is formed (Fig. 5c). Numerical simulation of the pressure waves at the top cross-section of the transducer (Fig. 5b) clearly shows a pressure profile consisting of two split regions near the center at two opposite phases; i.e., when one region is at the highest positive pressure and thus forms an optical waveguide, the other is at the highest negative pressure and cannot confine light. These two regions alternate between high pressure and low pressure states every half cycle. Therefore, by synchronizing the pulsed laser with one of the high-pressure regions, we can ensure that light is piped through that region and not the other. By flipping the relative phase between the two sets of elements, light can be switched over to the other waveguide (Fig. 5d, e). In this way, the formation of degenerate modes allows for digital steering of light from one spatial region to another through mode hopping.

We can also form more complex and reconfigurable patterns using the phased array by continuously changing the amplitude and/or phase. In general, our degrees of freedom are: the frequency of ultrasound, the laser pulse modulation, the relative phase between the laser modulation signal and the ultrasonic wave, and the phase and amplitude of each piezoelectric element. By changing the frequency of ultrasound, we can achieve different complex patterns of pressure waves in the region where the launched ultrasound waves from each element interfere and form a complex interference pattern. Also, by tuning the relative phase of the ultrasound excitation and the pulsed laser modulation, we can force light to interact with only a certain part of the ultrasonic standing waves. By changing the amplitude and phase of the piezoelectric elements we can reconfigure the pattern of pressure waves and as a result, the optical beam pattern can be dynamically reconfigured.

**Fig. 6:**
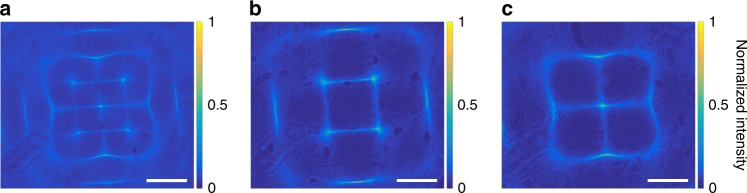
High frequencies excite higher order optical modes. Six elements (1, 2, 3, 5, 6, and 7) of the eight-segment array (same as Fig. 5a) are driven at a higher frequency of *f* = 1.216180 MHz to excite higher order modes. Experimental images showing that a continuous light (no modulation) and integration over time produces the superposition pattern (**a**) composed of the two modes in **b** and **c**. Note how the four spots around the center in **b** disappear and light couples instead to the center spot in **c**. The laser light modulation phase shift is **b** 0° and **c** 180° with respect to the ultrasound waves. The intensity of each 2D cross-section is normalized to its maximum value and the scale bar is 500 μm

As an example, if six elements (1, 2, 3, 5, 6, and 7) of the previously mentioned eight-segment array (same as Fig. 5a) are driven at a much higher frequency of *f* = 1.216180 MHz to excite higher order interference modes, a complex pattern of ultrasonic standing waves form that results in the optical beam pattern shown in Fig. 6a for a continuous laser. Different parts of the pressure profile are indeed out of phase. As a result, if we modulate the laser at *f * = 1.216180 MHz and synchronize it with the ultrasound wave, we can achieve completely different patterns of light by tuning the relative phases. For example, if the laser modulation and the ultrasound waves are synchronized with no phase difference, we can achieve the pattern shown in Fig. 6b, whereas we would achieve the pattern shown in Fig. 6c, if the phase difference is π. Note how the four spots around the center in Fig. 6b disappear and light couples instead to the center spot in Fig. 6c. The pattern in Fig. 6a is the superposition of these two patterns. By continuously changing the phase difference, we can continuously change the optical pattern between that of Fig. 6b, c. We can see from this example that by changing two of the parameters, i.e., the frequency of ultrasound waves to excite higher order modes and the relative phase between the ultrasound waves and laser light, we can sculpt complex patterns of light in the tissue.

### Ultrasound can guide light in mouse brain tissue

**Fig. 7:**
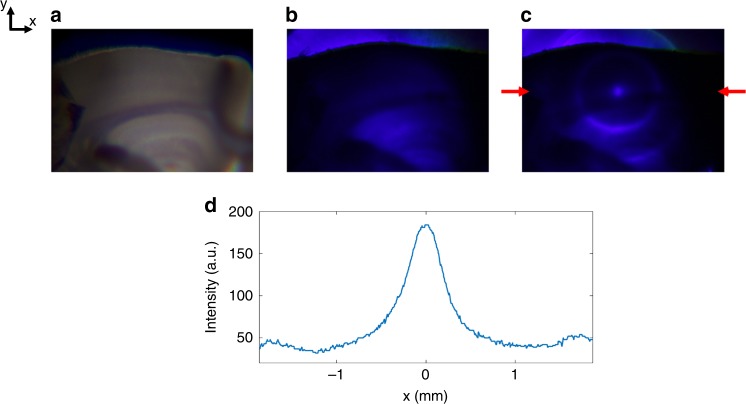
Light is confined within a mouse brain tissue. **a** Bright field microscope image of a 240 µm thick coronal brain slice from a mouse. **b** Blue light imaged through the brain slice tissue with no ultrasound. Laser beam is traveling along *z-*direction, impinging on the brain slice from the bottom and being imaged from the top, as shown in Fig. 2a. **c** Blue light imaged through the tissue couples to a sculpted waveguide, when ultrasound is turned on. **d** Line cross-section of the confined optical waveguide mode through the brain slice tissue near the top edge of the slice. The line-cut is through the line indicated by the red arrows in Fig. 7c. The measured extinction ratio (ER1) is ~5

To demonstrate the application of our proposed acousto-optic method for in-tissue light beam guiding, experiments were performed on mouse coronal brain slice tissues (Fig. 7a) (see Methods for details of brain slice preparation). We used a 240 µm thick tissue, which is a typical thickness of brain tissue used in slice electrophysiology. Blue light, as well as higher wavelengths in the visible spectrum, is particularly relevant for optogenetic stimulation protocols (e.g. λ = 480 nm)^35,36^. As wavelength decreases, scattering of light in tissue increases nonlinearly and absorption becomes dramatically larger^37^ The scattering and absorption properties of the mouse cortical tissue depends on the type, age, and region of the cortex. Different values in the range of 6.98–29.54 cm^−1^ at wavelengths *λ* in the range of 453–480 nm have been reported in literature for the reduced scattering coefficient of a mouse cortical tissue^38,39^. The reduced scattering coefficient increases as the wavelength is decreased. We used a blue laser at λ = 405 nm as an extreme case, which undergoes a large amount of scattering and absorption in the tissue (Fig. 7b). At this wavelength, the optical thickness for the cortical region of the 240 µm thick mouse brain tissue is larger than 7ℓs. Laser light impinges on the brain tissue from the bottom and the ultrasonic phased array is placed around the brain slice. Using our technique, light can be piped and collected at a spot using a virtual waveguide through the brain slice tissue thickness (Fig. 7c). A line cross-section of the optical waveguide in the brain tissue is shown in Fig. 7d, where it can be clearly seen that light is confined in the core of the waveguide. The measured extinction ratio is ER1~5. The ultrasonic steering of the optical beam at the boundary of different regions in the brain with inhomogeneous scattering and absorption properties would require careful adjustment of the ultrasonic phased array properties to compensate for the effects of the irregularities within the tissue on the ultrasonically sculpted light paths.

## Discussion

Our work demonstrates that patterned ultrasound can be leveraged to produce a variety of light piping components within the medium itself. Of particular interest is biological and neural tissue, where light techniques are fundamental to many sensing and actuation strategies, but penetration remains a problem. To be clear, this technique does not address absorption; instead, it plays the same role as waveguides do, i.e., confinement and guiding of light to counterbalance beam expansion due to scattering and diffraction. Our results in a homogeneous medium with negligible scattering show that ballistic photons can be confined and guided using the ultrasonically-defined optical waveguides. In a scattering medium both ballistic and scattered photons exist. While our experimental results demonstrate that this method works in a scattering medium, it would be important to study how the ultrasonically sculpted optical waveguide affects both scattered and ballistic photons in a turbid medium.

Additionally, the demonstrated technique does not require high average ultrasonic power. Both the ultrasound and the laser can be pulsed. The effective light-ultrasound interaction through the medium happens only during the tiny fraction of the ultrasonic wave period set by the modulation of the laser and the relative phase between the laser modulation and the ultrasound standing wave. In addition, different optical processes of biological interest respond to light over different time scales. For example, opsins integrate incoming photons over the course of milliseconds^35,36^; by comparison, the ultrasonic waves used here have periods in the microseconds. As a result, neither the light source nor the ultrasonic wave have to be continuously on but can be pulsed rapidly. For diagnostic and therapeutic applications, the established maximum values of de-rated spatial-peak pulse-average intensity (*I*_SPPA_), spatial-peak temporal average intensity (*I*_SPTA_), and mechanical index (*MI*) as defined by FDA are 190 W cm^−2^, 720 mW cm^−2^, and 1.9, respectively^40^. We performed ultrasonic power measurements outside the transducer to probe the tail of the ultrasonic pressure waves to obtain the maximum power required for realizing optical waveguides discussed in this paper (see Methods). For the extreme case, when all elements of the ultrasonic phased array are synchronously driven at 20 V, we measured *I*_SPPA_ = 50.365 W cm^−2^, *I*_SPTA_ = 503.7 mW cm^−2^, and *MI* = 1.246, respectively, at the highest pressure intensity region for a pulsed ultrasound at a duty cycle of 1%, (during the on time, the transducer elements are driven with approximately 40 cycles, corresponding to ~40 μs, with a pulse repetition frequency (PRF) of 250 Hz), which are all within the safety limit. The ultrasonic wave could be pulsed at much smaller duty cycles to reduce its thermal effect even further.

Reconfigurable optical pattern generation in tissue using this acousto-optic technique is not limited to the two examples demonstrated in this paper. One can imagine steering a single virtual optical waveguide simply by physically moving the array of ultrasonic transducers or selectively turning on and off individual transducer elements in a digital manner or continuously changing the pattern of light by continuously changing the phases and amplitudes of the array elements.

It appears likely that with the advanced ultrasound beam-steering technologies now in development for various medical applications such as High Intensity Focused Ultrasound (HiFU)^41–43^ complex pressure patterns can be created in tissue. Of course, when contemplating applications in the central nervous system, the ultrasound propagation loss in the skull can present a challenge^44^ and this will likely necessitate the use of craniotomy windows or burr holes to place an array of ultrasonic transducers on the surface of the brain^45,46^. Using our acousto-optic technique, ultrasound transducers can be placed outside the brain to launch ultrasonic waves from the surface to guide and steer light from a laser or light emitting diode through the craniotomy window. Initial simulation results (data not shown), indicate that planar, multi-element surface arrays (as opposed to cylindrically-symmetric arrays like those employed in this work) can transiently produce cylindrical pressure regions tissue similar to those demonstrated in this paper that extend into the tissue by beam-forming. With this approach, light can be guided, steered, and focused deep into the brain tissue without inserting any invasive light guide into the tissue. Lastly, while we demonstrated all of our results based on ultrasonic standing waves, there is also a great potential for using pulsed ultrasound to sculpt transient optical waveguides with arbitrary shapes and trajectories. Nevertheless, taken together, our results suggest the tantalizing notion that a variety of optical components can be instantiated within tissue.

## Methods

### Modeling and simulation

To model the ultrasonic waves generated using the cylindrical phased array, we used the Finite Element Method (FEM) implemented in a commercial FEM software environment (COMSOL, Inc.) using a coupled multiphysics simulation including solid mechanics analysis, electrostatics, and pressure acoustics.

Specifically, the Navier’s equation is solved for the piezoelectric structure, Gauss’ law for the electrostatics, and the Helmholtz equation for the pressure acoustics as follows:

Navier’s equation:

1 $$- \rho \omega ^2{{u}} - \nabla \cdot \sigma = {\boldsymbol{F}}e^{i\varphi },$$

where ***u*** is the displacement field, *σ* is the stress, ***F*** is the external body force, and *ρ* is the density of the solid, *e*^*iφ*^ is harmonic component, and *ω* is the temporal frequency.

Gauss’ law:

2 $$\nabla \cdot {\mathbf{D}} = \rho_\nu ,$$

where

3 $${\mathbf{D}} = \varepsilon {{E}}.$$

***D*** is the electric displacement, ***E*** is the electric field, *ρ*_*v*_ is the volume charge density, and *ε* is the permittivity.

Helmholtz equation:

4 $$\nabla \cdot \frac{1}{\rho_c }\left( {\nabla p_t - {{q}}_d} \right) - \frac{{k_{\rm eq}^2p_t}}{\rho_c } = Q_m,$$

where, *p*_*t*_ = *p* + *p*_*b*_ is the total pressure, *p*_*t*_ is the sum of perturbation sound pressure *p* and the background pressure *p*_*b*_, the parameter ***q***_*d*_ is the dipole source, $k_{\rm eq} = \sqrt {\left( {\frac{\omega }{c}} \right)^2 - k_z^2}$ is the equivalent wavenumber, containing both the ordinary wavenumber *k* as well as the out-of-plane wavenumber *k*_*z*_ and circumferential wavenumber *m*, *Q*_*m*_ is the monopole source, *ρ*_*c*_ is the density, and *c* is the speed of sound. The boundary electrical potential applied to the inner and outer walls of the piezoelectric elements generate an electric field that couples with the piezoelectric strain through a compliance matrix due to the piezoelectric material property. The harmonic strain, or equivalent vibrational displacement periodically changes the density of the surrounding fluid medium and generates ultrasonic pressure waves according to the Helmholtz equation above.

Both 2D and 3D simulations were performed. In the 2D case, we simulated a cross-section of the cylindrical geometry, assuming that it is infinite along the axial direction (*z*-axis). The 2D simulations were used as an approximate method to design our experiments for beam steering and complex pattern generation. We verified the results of our 2D simulations for a single element phased array (i.e., a cylindrical transducer) by comparing the results against an analytical solution^21^. We achieved agreement to within 0.18% between the numerically calculated resonance mode frequencies and the analytical results. For single element transducers, we performed 3D simulations. Since we are only interested in the cylindrically-symmetric modes, we reduced the simulation domain to the radial cross-section of the structure. In all our simulations, we used a perfectly matched layer (PML) boundary condition for the outer boundaries to mimic an infinite medium and prevent any reflection and interference of waves. We set the freely vibrating boundary condition to the inner and outer walls of the piezoelectric cylinder. The inner wall is held at the ground level potential and 20 V voltage is applied to the outer wall. In this arrangement, the polarization of piezoelectric cylinder occurs in radial direction.

The material and geometrical properties used in the FEM simulations are listed in Table 1.

**Table 1:** Material and geometrical properties used in the FEM simulations

*Piezoelectric transducer elements*	
Material	Piezoelectric PZT – 5 A
Density	7500 kg m^−3^
*Ultrasonic cavity (cylindrical)*	
Radius	19 mm
Height	30 mm
*Simulation domain (cylindrical)*	
Radius	*R*_out_ = 27 mm
Height	40 mm
Medium density (water)	1000 kg m^−3^
Speed of sound	1480 m s^−1^
*Perfectly Matched Layer (PML)*	
Radius range	*R* = 27–30 mm

We used an unstructured triangular mesh for all the different domains. To obtain converged results, we used a 0.2 mm maximum mesh size in the piezoelectric material region, a 0.15 mm maximum mesh size in the cavity and a 1 mm mesh size in the PML region.

We solved the set of coupled equations both as an eigenmode problem and also as a frequency domain problem. In the first case, all the eigenmodes were obtained simultaneously. In the latter case, we scanned the frequency in fine steps and monitored the pressure maximum in the center of the cavity to obtain the sustained cavity resonance modes. Using both methods, we obtained the same resonance frequencies. The frequency domain analysis helps with the understanding of pressure profile changes when sweeping the frequency to move from one mode to the next.

To obtain optical waveguide modes, the pressure profile is first transformed into a refractive index modulation profile in the medium^47^. The refractive index profile is used as the input to our finite difference mode analysis. We used a commercial-grade eigenmode solver (http://www.lumerical.com/tcad-products/mode/; Lumerical Inc.) to calculate the supported optical modes at a transverse cross-section of the sculpted optical waveguide. We used a non-uniform mesh structure with 30 nm mesh elements in the core of the waveguide and gradually increasing mesh elements along the radial direction to ensure the convergence of the results, while preventing numerical instability. The ultrasonically sculpted optical waveguides support several modes in the visible range of the spectrum. A selection of the supported modes is shown in Fig. 1d–g at *λ* = 650 nm.

### Optical setup and experimental approach

To image the formation of optical waveguides in the tissue as a result of interaction with the ultrasound waves, we built a vertical characterization setup consisting of a water tank, a laser source, a fixture to hold the ultrasonic transducer array, a fixture to hold the tissue, and a microscope assembly on top (Supplementary Fig. 1). Each fixture is mounted on an *xyz* stage equipped with precision micrometers (Thorlabs Inc., USA). The imaging microscope is made of a 5 megapixel CMOS camera (Blackfly S color 5.0 MP, Point Grey Research Inc., USA), a zoom imaging lens (VZM 600i, Edmund Optics Inc., USA) and a clear glass window lens acting as the interface between the microscope and the fluid in the tank. We used Thorlabs 30 mm cage system and also custom-machined parts to build the microscope column. The objective lens is immersed in the tank and is used to image cross-sections of the optical pattern in the tissue.

The tank is built from acrylic sidewalls and a high-quality quartz bottom to minimize distortion of expanded laser beam. The laser is mounted at the bottom of the tank. A beam expander is designed in front of the laser to expand the beam of laser to a 15 mm uniform spot. To couple light to the central waveguide in a fixed-position ultrasonically sculpted optical waveguide, we can use a narrow beam of light instead of an expanded beam, where the input beam can be optimized to match the NA and maximize the coupling efficiency.

The fixture to hold the transducer array is carefully designed and made from acrylic so that the contact surface area is minimized and no clamping force is applied to minimize the distortion of ultrasonic modes of transducers. Transducer elements are assembled with silver wires using silver paste. The whole array is then coated with an insulating Parylene C polymer using chemical vapor deposition (CVD) at room temperature (Specialty Coating Systems, USA). Input waveform generators (Keysight 33522B, Keysight Technologies, USA) and a power amplifier (ENI A300, Electronics & Innovation Ltd., USA) are used to feed electric pulses to the transducer array. The laser beam is modulated using a square wave at the same frequency of the ultrasonic waves. The laser modulation signal is phase locked with the ultrasonic waves so that the laser is turned on only when ultrasound pressure wave is at its maximum. This way we can synchronize the laser beam with the ultrasonic pressure pattern by adjusting the relative phase.

In each experiment, we sweep the input frequency in fine steps to obtain resonance conditions for the transducer array. Then the frequency is fixed and the phases are adjusted to obtain the waveguide pattern of interest. The integration time is always adjusted so that the maximum intensity of the acousto-optic waveguide is always below the saturation level for the CMOS camera.

### Tissue phantom preparation

To produce tissue phantoms with specific scattering properties, we used varying amounts of powdered bacteriological agar (A5306, Sigma-Aldrich, USA) and Intralipid 20% emulsion (I141, Sigma-Aldrich, USA). The powdered agar is first mixed in DI water at room temperature in a Pyrex beaker, covered by an aluminum foil to prevent evaporation. We use a concentration of 2 g agar in 100 ml of deionized water. Then the solution is constantly mixed with a magnetic stirrer at 600 rpm while the temperature is increased to the boiling temperature of the solution. When the solution is boiling, the hotplate is turned off and the temperature of the mixture monitored. Once it reaches 60 °C, we add the appropriate amount of Intralipid 20% (e.g., 1 ml for the 0.2% concentration), while continuously stirred at 800 RPM, to ensure that Intralipid is uniformly mixed in the solution. After 5 min, the solution is poured into a cylindrical mold to form a cylindrical phantom. The bottom of the tube is sealed with a smooth nonstick glass plate to ensure the facet of casted phantom is smooth. As the solution reaches room temperature, we move it to a refrigerator, where it is kept at 6 °C for 30 min. Prolonged exposure to air causes the molded phantom to shrink in size and become opaque. To prevent the shrinkage, we keep agar gel phantoms in deionized water after fully cured. Using this method, phantoms of varying Intralipid concentration and geometry can be created.

### Brain slice preparation

Animal tissue experiments were performed in accordance with protocols approved by Institutional Animal Care Use Committee at the University of California, San Francisco. Brain slices were prepared as described previously^48^. Briefly, Adult C57BL/6 mice (7–10 weeks old) were anesthetized with isofluorane and decapitated. Using a vibratome (Leica VT1200 S), coronal brain slices were prepared at different thicknesses (ranging from 150 μm to 3 mm) in a chilled slicing solution consisting of 234 mM sucrose, 11 mM glucose, 24 mM NaHCO_3_, 2.5 mM KCl, 1.25 mM NaH_2_PO_4_, 10 mM MgSO_4_, and 0.5 mM CaCl. Slices were then fixed overnight in 4% paraformaldehyde/1X PBS. Experiments were performed in artificial cerebrospinal fluid (ACSF) containing (in mM): 126 NaCl, 26 NaHCO_3_, 2.5 KCl, 1.25 NaH_2_PO_4_, 1 MgCl_2_, 2 CaCl, and 10 glucose and equilibrated with 95% O2 / 5% CO_2_.

### Ultrasonic safe operation

For diagnostic and therapeutic applications, the established maximum values of de-rated spatial-peak pulse-average intensity (*I*_SPPA_), spatial-peak temporal average intensity (*I*_SPTA_), and mechanical index (MI) as defined by FDA are 190 W cm^−2^, 720 mW cm^−2^, and 1.9, respectively^40^. In all of our experiments presented in this paper, the de-rated pressure intensity in the tissue is small enough such that always *I*_SPPA_ < 50.365 W cm^−2^ and MI < 1.246 at the highest pressure intensity region, which are both within the safety limit. In order to meet the requirements for *I*_SPTA_, we can design ultrasonic wave pulse train to have a small duty cycle (e.g., 1% duty cycle will result in 503.7 mW cm^−2^ < 720 mW cm^−2^), which ensures safe operation.

## Supplementary Information

Reporting Summary

Supplementary Information

## Data Availability

The data that supports the findings of this study are available from the corresponding author, M.C., upon reasonable request.
