# Supplementary material for: Ultrasonic sculpting of virtual optical waveguides in tissue
Source: Nat Commun. 2019 Jan 9;10:92. doi: 10.1038/s41467-018-07856-w (PMC6327026; doi:10.1038/s41467-018-07856-w)
Supplement: Supplementary file 2 — Supplementary Information [file 41467_2018_7856_MOESM2_ESM.pdf]

Supplementary Information

Ultrasonic sculpting of virtual optical waveguides in tissue

Maysamreza Chamanzar *et al.*

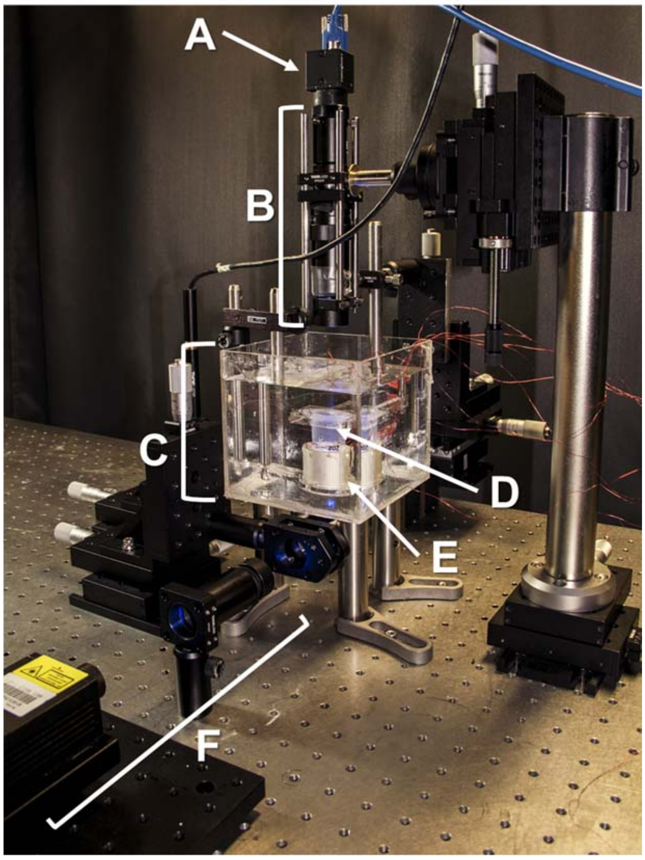

Supplementary Fig. 1 Experimental setup consisting of a custom-built microscope, a container to hold the tissue and ultrasonic phased array in the solution (ACSF, water, etc.). Two fixtures are designed attached to two independent precision xyz stages. One of them is used to hold the transducer array, and the other one holds the tissue. Laser beam is aligned to impinge on the tissue from the bottom of the container. A beam expander is designed in front of the laser to expand the beam. Different elements of the setup are: A – CMOS camera, B – Zoom lens C – Container, D – Dropdown fixture to hold the tissue phantom, E – Transducer array, F – Laser assembly.

Supplementary Tab. 1 Optical properties of scattering tissue used in this study. The tissue phantoms were calibrated using a standard Oblique Incidence Reflectometry (OIR). We have assumed a typical anisotropy factor of g = 0.9 (ref. 31).

**Sample**	**$\mu s'$ (cm^{-1})**	**Thickness (µm)**	**ℓs (µm)**	**Optical Thickness (ℓs)**
2% Agar + 0.2% Intralipid	2.35	8000	425.53	18.80
Mouse cortex (λ = 453 - 480 nm)	6.98 - 29.54 (refs. 38 - 39)	240.0	33.85 - 143.27	1.68 - 7.09
